# Strategic partnership for Health in All Policies and sustainable transport in Scotland: a case study evaluation

**DOI:** 10.1016/j.puhip.2025.100624

**Published:** 2025-05-14

**Authors:** Margaret J. Douglas, Anna Gale, Rok Hrzic, Timo Clemens, Adrian L. Davis

**Affiliations:** a Department of International Health, Care and Public Health Research Institute – CAPHRI, Maastricht University, the Netherlands; b Public Health Scotland, Gyle Square, 1 South Gyle Crescent, Edinburgh, EH12 9EB, UK; c School of Health & Wellbeing, 90 Byres Road, University of Glasgow, G12 8TB, UK; d Edinburgh Napier University, Transport Research Institute, 10 Colinton Road, Edinburgh, EH10 5DT, UK

**Keywords:** Health in all policies, Intersectoral collaboration, Healthy public policy, Transport, Social determinants of health

## Abstract

**Background:**

Health in All Policies aims to ensure policy decisions across sectors improve health and health equity. Principles of a Health in All Policies approach have been defined as Governance, Comprehensive approach to health, Collaboration, Equity, Participation, Evidence-based and Sustainability. Intersectoral partnerships are a recognised mechanism for Health in All Policies but few evaluations study partnerships that aim to influence policy. This case study evaluation studied a national Partnership focused on transport policy in Scotland. The evaluation aimed to assess the extent to which the Partnership meets the principles of Health in All Policies and informs policy and practice. It also identified actions to improve its impact.

**Study design:**

Anonymous self-completion survey of members of the Partnership and its wider Learning Network.

**Methods:**

The survey used Likert scales to assess respondents’ views on whether the Partnership was meeting its aims and supporting principles of Health in All Policies. Respondents also recorded whether the Partnership had increased their knowledge, supported wider collaboration or informed decision making. The Partnership used structured discussion in groups and an online poll to generate and prioritise improvement actions.

**Results:**

A vast majority of respondents scored the Partnership highly for Comprehensive approach to health (82 %), and being Evidence-based (78 %). Most rated it highly for Governance (63 %), Collaboration (62.5 %) Equity (63 %) and Sustainability (57 %). However, less than half (43 %) scored it highly for Participation. Respondents indicated a range of ways the Partnership impacted on their knowledge and practice. The top actions identified by the Partnership to improve its impact were to investigate car culture and identify specific national transport policies to influence.

**Conclusions:**

A national sector-specific Partnership can provide a constructive platform for a Health in All Policies approach to improve health and health equity, but further mechanisms are needed to support participation of affected populations.

## 1 What this study adds

• This case study shows that a national sector-specific Partnership can support productive collaboration between public health and other sectors and meet most of the principles of a Health in All Policies approach.
• The study highlights the challenge of gaining meaningful participation of affected populations in partnerships working at a national level.

## 2 Implications for policy and practice

• A similar partnership model could support collaboration and influence policy in other settings and other sectors.
• Other mechanisms and approaches are also needed to support community participation in Health in All Policies.

## 3 Introduction and background

Health and health inequalities are shaped by policies and activities across many areas of life [1]. This requires public health professionals to work across sectors to influence wider policies for better health outcomes [2]. Health in All Policies (HiAP) is *‘an approach to public policies across sectors that systematically takes into account the health and health systems implications of decisions, seeks synergies and avoids harmful health impacts, in order to improve population health and health equity‘* [3]. HiAP involves working closely with planners and policy makers to inform policy making, often using specific processes like health impact assessment (HIA) [4]. Principles to underpin HiAP have been defined as: Governance, Comprehensive approach to health, Collaboration, Equity, Participation, Evidence-based and Sustainability [4].

Road transport is an important sector for HiAP, because transport policies affect multiple determinants of health and health inequalities [5]. Active transport modes provide health benefits [6] including physical activity [7], social interaction [8], improved mental health [9], footfall for local businesses [10] and increased perceived safety [11]. Conversely, private car travel and car dominated environments have adverse health impacts [12] through air and noise pollution [13], road injuries [14], physical inactivity [15], the severance effect of heavy traffic [16] and financial hardship from ‘forced car ownership’ [17]. Transport also affects access to other essential health determinants [5]. Good public transport can prevent ‘transport poverty’ - a lack of transport options that are available, reliable, affordable, accessible and safe – affecting health and health inequalities [18].

Intersectoral collaboration is central to HiAP and can involve informal relationship building and/or formal structures such as inter-departmental committees, cross agency groups and partnerships [19]. Literature highlights the challenges of intersectoral working and lack of evidence of its effectiveness [20]. Examples exist of partnerships focused on healthy public policy [21,22]. A realist evaluation of European Healthy Cities Phase V found that intersectoral partnerships implementing HiAP can influence policy [[23], [24], [25]]. However, most evaluations study partnerships aiming to develop community and/or organisational capacity rather than policy impact [[26], [27], [28]]. Studies of partnerships seeking to influence policy often involve lobbying and campaigning rather than collaboration with policy makers [29,30]. Less research explores inter-sectoral partnerships focused on influencing policy rather than delivering projects or community capacity.

The Public Health and Sustainable Transport (PHST) Partnership is a national partnership focused on links between transport policy and health in Scotland [31]. Members include transport policymakers and professionals in national and local government, third sector sustainable transport organisations, academics and public health professionals. It aims to: collate evidence of the benefits and harms of transport policies; articulate their impact on health and inequalities; and inform national and local policy and practice.

The Scottish National Transport Strategy identifies ‘Improves our health and wellbeing’ as one of four priorities [32]. However, until the PHST Partnership was established in 2020 there was no national group focused on health and transport. The Partnership has conducted HIAs of national transport policies for: reallocation of road space [33], the route map to reduce car km [34], and active travel guidance [35]. It has published a report on transport poverty [18] and is developing transport poverty indicators.

In 2023 the Partnership established a Learning Network for anyone in Scotland working in transport, public health or a related sector. This aims to link transport and public health professionals at local levels and provides seminars, training and discussions through an active Teams site.

This paper aims to contribute to research on collaborative partnerships focused on influencing policy, by reporting a case study evaluation of the PHST Partnership. It aimed to evaluate members’ views on the extent to which it was meeting its intended aims, fulfilling the HiAP principles and informing policy, knowledge and practice, to inform actions to improve its work.

## 4 Methods

The evaluation involved an anonymous self-completed online survey using the Lime Survey platform, in March 2024. The survey asked about respondents’ backgrounds and membership of PHST sub-groups. Respondents used Likert scales from 0 to 5 to score: how well they think the Partnership is meeting each of its aims and supporting each HiAP principle (Governance, Comprehensive approach to health, Collaboration, 10.13039/100028163Equity, Participation, Evidence-based and Sustainability [4]). Yes/no responses indicated if the Partnership enabled respondents to: increase knowledge; support advocacy; make new connections; collaborate with others; or use the outputs to inform decision making. The questionnaire invited free-text comments, noting that care would be taken to avoid sharing identifiable details. It was piloted with three Partnership members before being finalised. The survey invitation was circulated using the group mailing lists and Teams site.

The aggregated number of responses to each question were presented, with scores of 4 or 5 considered to be high.

Free text responses were extracted separately for thematic analysis. Two authors (MJD and AG) reviewed all comments to identify themes independently, then compared these to agree final themes by consensus.

Survey findings were presented at a Partnership meeting and a structured process was used to identify future work priorities. This involved four facilitated small groups discussing findings and identifying actions to improve the work. In plenary, each group suggested actions in rounds, one suggestion at a time per group. Similar suggestions were combined and the rounds continued until all the actions were collated in a consolidated list. Partnership members then voted on their top three priorities from the list of actions through an online poll.

## 5 Results

### 5.1 Respondents

A total of 580 people were invited to complete the survey, including 60 members of the main Partnership and/or its Data and Evidence subgroup and 520 who were only on the more recently established Learning Network. Following reminders, the response rate was 14 % overall (n = 82) but 43 % among members of the main Partnership and/or Data and Evidence Subgroup (n = 26). Table 1 shows respondents’ backgrounds and group membership. Most are transport or public health professionals working in local government or the NHS.

**Table 1:** Respondents’ backgrounds and membership of PHST groups.

	n^a^
**Employer**	
National Government	8
Local Government	23
NHS	34
Other Public Sector	3
Third sector	10
Private sector	4
Academia	1
Other (retired)	1

**Work area**	
Transport	37
Spatial planning	10
Public Health	29
Health (other than public health)	8
Other (community learning, community planning, poverty, resilient communities, social impact, sustainability, employability)	9

**PH&ST group**	
Public Health and Sustainable Transport Learning Network	50
Public Health and Sustainable Transport Learning Network Steering Group	8
Public Health and Sustainable Transport Partnership Group	20
Public Health and Sustainable Transport Data and Evidence Group	13

a Respondents could select multiple responses for each.

### 5.2 Extent to which PHST is meeting aims and HiAP principles

**Fig. 1:**
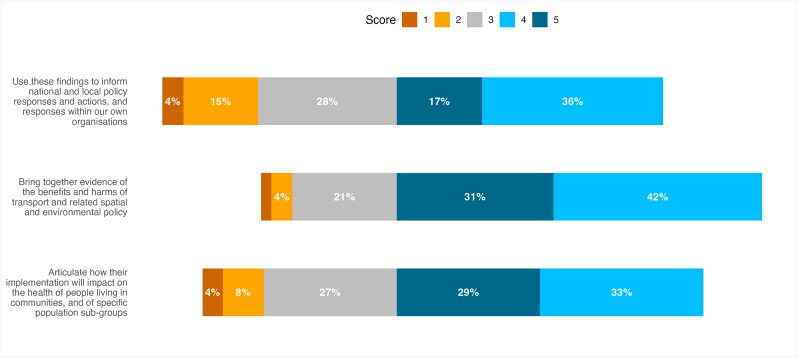
Respondents' scores for extent to which Partnership is meeting aims.

**Fig. 2:**
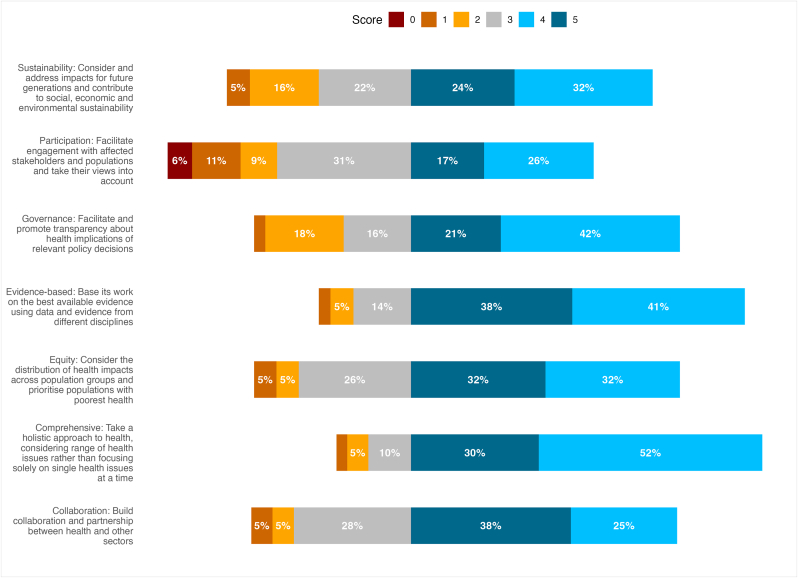
Respondents' scores for extent to which Partnership is meeting HiAP principles.

Respondents scored the extent to which the Partnership was meeting its stated aims and HiAP principles. Fig. 1, Fig. 2 show the spread of scores for the aims and principles.

These show that over 70 % of respondents scored highly (4 or 5) for the aim related to bringing together evidence (73 %), and for the principles of Evidence-based (78 %) and Comprehensive approach to health (82.5 %). Over 60 % scored highly for the aim to articulate health and inequalities impacts of transport policy (61 %), and the principles of Governance (63 %), Equity (63 %) and Collaboration (62.5 %). Just over half scored highly for the Sustainability principle (56 %), and for the aim to inform policy (53 %). Less than half (43 %) of respondents scored highly for Participation of affected populations. Scores were higher among members on the main Partnership and/or Data and Evidence Subgroup than those only on the wider Learning Network, but small numbers precluded formal significance testing.

### 5.3 Impact on knowledge and practice

Table 2 shows the numbers of respondents reporting that their involvement with the Partnership had impacted on their knowledge and practice. Thirty-nine people reported increased knowledge of links between transport and health, 28 reported new professional connections, and 33 reported using new resources or approaches including HIA, public health websites, data dashboards and the Place Standard Tool [36].

**Table 2:** Number of respondents reporting impact on knowledge and practice.

Reported impact arising from involvement in the groups	n
Increased your knowledge and understanding of the relationship between health and transport	39
Gained attention to support advocacy	9
Made new professional connections and contacts	28
Collaborated with colleagues in other sectors	19
Used outputs and connections from the group to inform decision making in my own organisation	18
Used outputs and connections from the group to influence decision making in other organisations	13
Used specific resources or approaches to inform policy or practice	33
Others: • Informing the development and content of work to influence wider agendas • Use information in project work • Used outputs to present locally on transport poverty	

### 5.4 Survey free text responses

Twenty-one respondents provided free text responses. From these we identified five main themes: Strength of collaboration; Learning and resources; Wider engagement and messaging; Effecting change and demonstrating impact; and Complicated structure. These are described with illustrative quotes below.

#### 5.4.1 Strength of collaboration

This was the strongest theme, with multiple respondents commenting positively on the multi-disciplinary nature of the Partnership and the strength of collaboration. A few suggested other interests to involve, including transport operators and representatives of other population groups.

‘I think this is an extremely valuable model of engaging and working with partners and stakeholders to bring a HiAP approach. I have learnt an enormous amount for engaging directly with stakeholders to develop and produce evidence-based work’.

#### 5.4.2 Learning and resources

Many respondents identified shared learning arising from the Partnership. They identified several specific activities contributing to this including training, webinars, discussion forums and health impact scoping exercises.

‘Membership of the group has been of invaluable assistance to us connecting with the public health agenda’.

#### 5.4.3 Wider engagement and messaging

While recognising the collaboration within the Partnership, several respondents also identified a need for wider engagement beyond the Partnership. They suggested extending engagement to further stakeholders, decision makers and the public. Some noted a need to reach local actors who face competing pressures.

‘Wider, deeper, and fuller engagement and delivery is required across all stakeholders, and the public’.

‘I am always mindful about how outputs will land at a local level. The complexity is enormous, and not all opportunities are taken at a local level to progress action on this important building block of good health due to limited resource’.

#### 5.4.4 Effecting change and demonstrating impact

Many respondents commented on the Partnership's impact on policy and practice but there were mixed views on the strength of impact. Some reported that the Partnership's work had influenced policy and/or practice in their own work or organisation, but several others suggested that further actions are required to influence policy. This linked to the ‘wider engagement and messaging’ theme, with suggestions that wider engagement was crucial to understand the levers for change and achieve broader impacts on decision making.

‘I have really enjoyed the material provided through the networking group and I was able to use it in my project work. I am not sure the message resonates with the general population though’.

‘I think we are good at discussing matters and building an evidence case. Maybe we need to think about how we better get that content out more and influence dissenting voices’.

#### 5.4.5 Complicated structure

Finally, a few respondents commented that the large size and the Partnership structure with multiple groups hindered understanding of roles. Most of these comments mentioned that they were new to the Partnership. Suggestions to address this included a register of members, a conference and in-person meetings.

‘As a newbie to the group, the one comment I would make is that it all feels a bit complicated with the different groups, meetings and activities going on’.

### 5.5 Partnership identification of priority actions

The small group discussions identified reflections and potential actions. Some groups expressed surprise at the relatively low score for the aim of informing policy, giving examples where they felt the Partnership had been influential. The groups noted the low score for Participation of affected populations and that it is difficult for a national partnership to involve public members directly. Finally, the groups identified that car-dominated culture and discourse hindered implementation of policies to reduce car use in favour of more sustainable modes.

The groups identified 14 possible actions in total. Twenty-three members completed the online poll to select their top three actions. Table 3 shows the identified actions and votes for each. The top action is to explore car culture, reflecting Partnership discussions on this topic. Other actions were to identify priority areas to influence, link to other government priorities and keep a rolling workplan of policies to engage with.

**Table 3:** Priority actions identified by Partnership members.

Action	Votes
Car culture – Workshop/paper on what led to current position, how to change, implications for health and equity	14
Set priority areas to influence	13
Make explicit link to show how priorities of group link to First Minister's priorities	7
Workplan of transport policies and topics we have engaged in with rolling note of actions and how we move forward with/develop partner consultations e.g. HIAs	6
Evaluate how we are informing policy and measuring our impact in this area	6
Broaden representation, e.g. Poverty Alliance – both population groups; regionally (RTPs) and providers.	5
Develop short life subgroups for key action-focused workstreams	5
Re-visit how to facilitate engagement of those experiencing transport poverty into policy	3
Re-visit purpose – why is it that we're doing well in some areas and not others – is it shifting priorities for the group? Or do we need to refocus efforts	2
Develop a communication strategy for media and wider public	2
Celebrate the work of the partnership and share with others	2
Engage with politicians	2
Carry out a needs assessment of the learning network	1
Review the format of the meetings, i.e. updates prior to meeting; use of meeting time to have breakout rooms	1

∗each respondent could vote for 3 actions.

## 6 Discussion

### 6.1 Summary of findings

The survey of members of a national multi-agency Partnership and Learning Network found that its members value the collaboration and opportunities for shared learning and score it highly for taking an evidence-based and holistic approach to health. However, it is not achieving participation of affected populations. The scores also suggest a need for better consideration of distributional and sustainability impacts, and further action to enhance policy impact. The Partnership identified priority actions, including investigating how to address car culture and identifying priority policies to influence.

### 6.2 Insights from theory and other literature

WHO recommends intersectoral partnerships as a key mechanism for HiAP [19] but other studies have focused more on mechanisms to support partnership rather than alignment with HiAP principles [26,27]. The findings highlight some key areas of improvement for the PHST Partnership, that could usefully be informed by other literature. The first is that to date the Partnership has lacked participation of affected populations. Public participation in public health is advocated to increase empowerment and reduce health inequalities but usually involves community level activity [37]. It may be more challenging to achieve meaningful participation at national level. Several authors have identified the challenges of community participation in HIAs and related work and the potential for tokenistic involvement [[38], [39], [40]] Other authors, like some Partnership members, have questioned whether meaningful community participation is feasible in partnerships at national level [20]. However, finding ways to involve affected populations in the Partnership's work could have multiple benefits. For example, community participation in HIAs provides useful evidence and insights [41], increases perceived agency in communities [42] and increases influence on decision making [42,43]. Wider participation could also help to reach the ‘dissenting voices’ that survey respondents identified. The Healthy Cities evaluation found that participation was supported by, and supported, overall governance [23]. Literature and guidance suggest ways to enhance participation [41], including stakeholder mapping, involving community organisations, assessing their readiness to engage and adapting methods depending on the context [44,45].

A second area highlighted is achieving policy influence. The Partnership uses HiAP mechanisms including HIA, policy review and shared data, and the collaboration involving policy partners should ensure the work remains relevant. However the survey scores and comments highlight the challenge of influencing policy and practice across national and local levels.

Previous studies have highlighted the importance of political support to embed health in policymaking [22,23,46]. Further insights can come from policy theories, which have been used to research and inform HiAP activities elsewhere [[47], [48], [49], [50]]. It has been argued that theories should not be used instrumentally but can help understanding of complex policy processes, avoid undue focus on technical solutions and help close the ‘expectation gap’ [51]. The Advocacy Coalition Framework is a theory that may help explain the PHST Partnership's place in a wider transport policy subsystem. It describes coalitions of actors who interact and compete [52,53]. Actors seek to translate their beliefs into action, including ‘deep core beliefs’ reflecting fundamental values, ‘policy core beliefs’ about how these may be realised in a particular sector and ‘secondary beliefs’ about practical implementation, which are more susceptible to change [52,53]. Evidence may be interpreted differently depending on these beliefs. The theory suggests policymaking tends towards continuity. Incremental changes may occur due to learning within coalitions but internal or external ‘shocks’ can provide a window of opportunity for significant shifts [52]. The Covid-19 pandemic was a shock that led to the PHST Partnership being established to address the impacts of the pandemic on transport and health. The theory suggests that the Partnership should be aware of competing coalitions opposed to sustainable transport and be alert to ‘shocks’ that may stimulate (either positive or negative) policy change.

### 6.3 Strengths and limitations

A strength of this work is the focus on principles of HiAP, using a bespoke questionnaire which was piloted before being finalised. The Partnership used a structured process to develop and prioritise future actions. However, limitations include the low response rate despite reminders encouraging response. The Learning Network was new and growing rapidly at the time of the survey so many network members had had limited involvement, likely affecting their response rate and responses. Small numbers precluded significance testing of differences between subgroups. The free text responses were short and so cannot provide very rich data to understand partnership dynamics but showed consistency in the themes. The authors involved in thematic analysis are Partnership members, which may have coloured our findings. However, we independently identified almost identical themes. Finally, as a case study evaluation, caution should be applied when generalising findings to other contexts.

### 6.4 Implications for policy and practice

The PHST Partnership is the only national level collaboration we know of that uses HiAP specifically for transport policy and reflects a positive policy environment. This evaluation has identified the value of partnership for collaboration across sectors and also some challenges. A similar model may be useful in other settings and other sectors. However, success factors and challenges may vary in other contexts, for example regional rather than national partnerships may work better for larger nations.

The Partnership has used the findings to inform its workplan and is collecting evidence to monitor its impact on policy and practice. We will also repeat the survey in future to monitor progress. Several of the respondents’ suggestions have already been implemented. These include holding a national conference, online in November 2024, and in-person regional meetings in early 2025. To improve participation of affected populations, opportunities for involvement of communities and wider interests are being considered through the Learning Network. The relatively low score for the Sustainability principle was surprising given the synergy between reduction of carbon emissions and positive impacts for health and health equity. The Partnership aims to express those links more clearly and is contributing to transport sections of the Scottish Government Climate Change Plan [54] and Just Transition sector plan [55] in 2025.

### 6.5 Implications for research

This study aimed to fill a research gap by evaluating a collaborative Partnership that aims to influence policy. Further research on similar partnerships is needed to understand links between context, mechanisms and policy outcomes. The survey could be replicated to compare with other partnerships and can be repeated to see future change. Qualitative research could usefully explore the dynamics of partnership collaboration and the enabling and inhibiting factors influencing its work. Further research could use a theory-informed approach to assess the policy impact of this and other partnerships.

## 7 Conclusion

This study found that members of the PHST Partnership scored it highly for many principles of a HiAP approach but identified challenges in enabling the participation of affected populations. The Partnership has used the evaluation to inform its workplan. Findings suggest that a national sector-specific partnership can provide a constructive platform for a Health in All Policies approach to improve health and health equity.

## Ethical approval

The study received a favourable ethical opinion from Public Health Scotland Ethics Committee (PEC) in March 2024. Reference number: PHS2023-24H028.

## Funding

This research did not receive any specific grant from funding agencies in the public, commercial, or not-for-profit sectors.

## Declaration of competing interest

The authors declare the following financial interests/personal relationships which may be considered as potential competing interests:MJD chairs the PHST Partnership group, ALD chairs the Data and Evidence subgroup and AG chairs the Learning Network Steering Group. We have no other competing interests to declare.
